# Positioning spice tourism as an emerging form of special interest tourism: perspectives and strategies

**DOI:** 10.1186/s42779-021-00086-4

**Published:** 2021-07-05

**Authors:** Bipithalal Balakrishnan Nair, Patita Paban Mohanty

**Affiliations:** 1 Sol International Hospitality Management (SIHOM), Woosong University, Daejeon, Republic of Korea; 2 School of Hotel Management, Siksha ‘O’ Anusandhan University, Bhubaneswar, Odisha India

**Keywords:** SIT, Heritagization, Rural sustainability, Spice tourism

## Abstract

The COVID-19 pandemic has halted activities in the global tourism industry, and the situation has only been worsened by the general air of uncertainty and lack of effective vaccinations. Consequently, people have begun testing various remedies to enhance their immunity, primarily turning to traditional medical practices and home remedies. The medicinal use of spices, given their immune-boosting properties, is increasingly popular globally and has enhanced global awareness of spices and their products. In light of this surging popularity, this study examines spice tourism as a concept of niche tourism. This study proposes spice tourism as a valuable post-COVID-19 strategy by providing four different approaches to position spice tourism within special interest tourism. This paper also suggests a tourism development plan for spice tourism and proposes a strategy for its resilience post-COVID-19.

## Introduction: Role of food in tourism

As a significant tourism resource, food is firmly related to tourism in several ways, in that food strengthens and is key to the visitor’s experience. Food has played a leading role in tourism decision-making and experience, tourism goods, and lobbying campaigns, and it can act as a valuable tool for destination development. Thus, tourists and the tourism sector around the world share valuing cuisine. However, specific issues require resolution to ensure that guest standards are fulfilled and even surpassed [1]. Food has become an evolving topic in the context of tourism and recreational activities and its flexible applicability as part of the community culture that tourists consume, the commercialization of commodities to express sustainable development, identity, and historical and ethnic characteristics goods services [2]. The role of food in tourism ranges from a wide variety of food uses as sustenance, as marginal to the visitor experience, and as the main attraction and motivation for traveling. Therefore, functional categorization of the role of food in tourism might make that role more decisive.

Cuisines act as both a push and a pull factor of travel motivation, on the one hand, “pushing people away from their familiar foods and eating patterns and pulling them toward new and exciting foods” [3]. However, this explanation further requires a destination-specific analysis to explain whether the place’s food acts as a pivotal or a supporting supply [4]. Principal resources are the core motivators in attracting visitors to a vacation spot—assets that support and strengthen the key motivating factors, although they may not serve as visitor-motivating factors. Thailand, France, and Italy are examples of countries that regard food as a critical resource [5].

Moreover, a nation’s cuisines are an integral part of destination branding, marking, and acting as factors in the destination’s distinctiveness. This cultural significance of food often creates identity and image for many destinations—for instance, kimchi in South Korea, samosa in India, and pizza in Italy. Ethnic food and cuisine could be a great tourism draw for rural destinations; consuming regional cuisine also could play an essential role as a leisure and cultural practice [6].

Related research covers the spectrum of food-based tourism experiences across several product categories, such as speciality tourism, agritourism, culinary tourism, food-tourism destinations, and incentive schemes to purchase food [7]. Promoting food-related practices has equal advantages for both the farming and the tourism industries of a single destination. Local food improves and strengthens the tourism commodity, while tourists provide a platform for extending and growing local food products. The increased competency in global tourism and the significance of specific food-tourism resources are the reasons for the emerging trends in food tourism. Although “food and tourism are part of a systemic network of production; tourism alone cannot increase the value of quality food” [8]. Therefore, based on their biodiversity, ethnic cuisine, and specialities, destinations are busily developing more innovative products. The many examples of this trend include wine tourism in New Zealand, tea tourism in India, beer tourism, pizza tourism, and kimchi tourism. Nonetheless, all these types require good posting and promotion to reach their maximum potential.

Food tourism forms, such as tea, coffee, beer, or spice tourism, are promoted with their specificities. For example, tea tourism is connected with tea diplomacy, concerning tea’s presence and popularity in diplomatic meetings and conferences. Similarly, this paper tries to position spice tourism as concerning the health benefits of spices. Compared to other food tourism, spice tourism is currently an unresearched area, yet with great potential for becoming a top attraction. Thus, the development of synergy and the introduction of efficient marketing approaches to enhance regional food items and tourism have enabled regions to grow into unique destinations [9]. Spices have functional applications, historical complexities, and the surrounding climate, and they include complex values and norms. Spices are used as a part of food and health and well-being, to be developed in various ways to add to spices’ cultural identity and be expressed in foodstuffs. Spice tourism is described as connected to history, development, consumption, and tourist experience within and around cuisine’ in places and attractions [10].

## COVID-19 and tourism new normal

Given the international health crisis resulting from the COVID-19 pandemic, concerns are being raised about its short-term and long-term impacts on the tourism sector [11]. Changes induced by the pandemic are likely to have substantial impacts on mobility, socialization, consumer behavior, recreation, and many other social dimensions of tourism. Despite this, Jurado-Almonte et al. [12] maintained that the tourism sector is highly resilient and can adapt to and recover from disastrous or unexpected events such as this. Other analysts believe that society will gradually return to some degree of normalcy once the worst moments of the pandemic pass. Ioannides and Gyimóthy [13] opined that every crisis opens some new opportunities. For instance, the “new normal” in tourism could integrate forms of resilience and reformation that are more sustainable, buoyant, intentional, and collaborative.

**Fig. 1:**
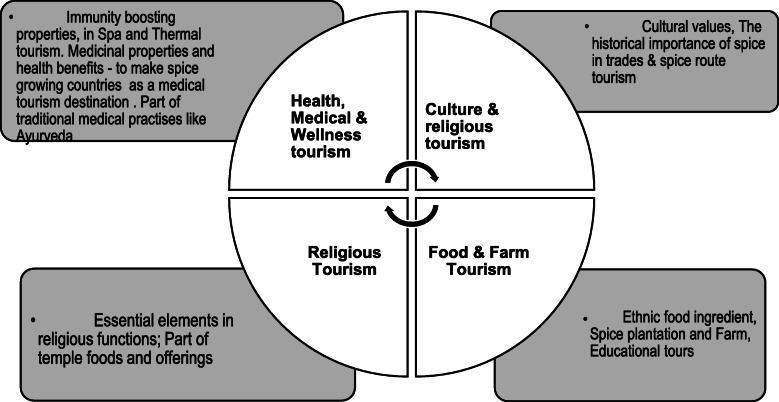
Conceptualization of spices as a tourist attraction

To this end, the COVID-19 pandemic has also appeared to (re)create demand for several forms of special interest tourism (SIT) is predicted to grow as a trend. These changes can be both positive and negative. As an example, while COVID-19 troubled cruise tourism, luxury tourism is precited to be a trendsetter. Similarly, spice tourism could be considered an emerging sector of SIT post-COVID-19. Spice tourism could be expanded due to spices’ immunity-boosting properties—specifically Indian kitchen spices such as turmeric, cardamom, fenugreek, garlic, and pepper. However, compared to other forms of plantation or food tourism, spice tourism is relatively unexplored within tourism landscapes and is still in its infancy as a topic of study [14].This moment could be an opportunity for spice-growing countries such as India, Sri Lanka, Guatemala, Mexico, and Jamaica to develop their future tourism strategies further. Accordingly, this study aims to (a) locate spice tourism in the broader framework of medical, health, food, and agricultural tourism as a specific type of special interest tourism (SIT); (b) examine the significance and future potential of spice tourism, and (c) analyze various avenues of product development in spice tourism. This study hopes to considerably enhance the current understanding of spice tourism within the realms of SIT and sustainable tourism development and gains different perspectives (Fig. 1).

## Positioning spice tourism within special interest tourism

If adequately marketed and developed, spice tourism could become one of the most prominent special interest tourism (SIT) forms globally. Rural agrarian efficiency is facing severe difficulties due to the pandemic, and this could help revive the tourism sector in spice-producing nations with substantial rural facilities in the developing world. The following section describes four approaches to develop spice tourism within the current industry.

### Approach 1: Medical tourism

Despite rapid advances in technology and research, spices have maintained their value in both the culinary and pharmaceutical industries. Many spices have antimicrobial, anti-inflammatory, and anticarcinogenic properties [15–19]. These properties might explain why spices are more widely used in tropical areas that experience more infectious diseases, and why they are so commonplace in the meat industry, which again is especially susceptible to spoilage problems.

Today, herbs and spices are consumed as functional foods. A World Health Organization (WHO) survey found that 70–80% of the world population depends on modern medicine sourced mainly from herbs for primary healthcare [20]. Likewise, 80% of developing countries and up to 60% of the global population depend directly on herbs and plants for their medical benefits [21]. The first scientific research on the use of spices as preservatives was presented in the 1880s, demonstrating the antimicrobial properties of cinnamon oil against Bacillus anthracis spores [22]. Furthermore, spices are a common food additive worldwide, enhancing the organoleptic properties of food and increasing shelflife by fighting foodborne pathogens [23]. Moreover, spices contribute a great deal to the medical, chemical, medicinal, perfumery, and food science industries.

In food and beverages preparation, spices can be used whole-dried, powdered, or as a paste. Spices are an essential component of human nutrition and contain essential proteins, fibers, fats, essential oils, minerals, and pigments, apart from bioactive compounds such as phenolic acids, sterols, and pungent substances piperine, and capsaicin (see Table 1). Spices like clove, cinnamon, black pepper, turmeric, and carom seeds effectively battle pathogenic bacteria. Spices have been used in cooking and medicine for hundreds of years [29]. Spices are extracted from medicinal plants and help cure infections, disease, and improve consumers’ overall health benefits. These medicinal spices are often referred to as herbal or phytomedicine and are derived from the seeds, root, bark, fruit berries, aril, pods, and flowers of various plants [30].

**Table 1:** Health benefits of spices

Common name	Botanical name	Health benefits
Turmeric	*Curcuma longa*	Possesses anti-inflammatory cum antimutagenic, antimicrobial and anticarcinogenic properties [15, 16]; reduces total cholesterol and LDL [17, 18].
Garlic	*Allium sativum*	An antioxidant that supports the body’s defense processes against oxidative damage and the common cold; reduces blood pressure and risk of heart disease, increasing testosterone in the testicles [24]; a natural antiseptic with antiviral and antidiabetic properties [25].
Cinnamon	*Cinnamomumverum*	Possesses antioxidant, antifungal, antibiotic, antimicrobial, and antiviral properties, and is even effective against some drug-resistant fungi; fights diabetes and reduces heart disease is anti-inflammatory, anticarcinogenic, and antidiabetic [26].
Black pepper	*Piper nigrum*	Boosts immunity, increases the concentration of good cholesterol, is antioxidant and anti-inflammatory, and treats sore throats due to flu [25]; enhances digestive tract function.
Cloves	*Syzygiumaromaticum*	Promotes antiviral and antimicrobial activity, is a hepatoprotective antidiabetic, and is an anti-inflammatory; reduces stress, is chemo-preventive, and acts as a total antioxidant; treats bad breath [27]; used as treatments for oral health, obesity, osteoporosis, COPD, coughs and colds, and vomiting; antiseptic; good for eyes and abdominal pain [28].

Spices have incredible health benefits both in raw and processed forms. Spices have various functions such as antioxidant, antiviral, and antimicrobial properties, because of these characteristics’ spices are integral parts of many forms of health and wellness tourism. For instance, Ayurveda (an ancient medical practice originated in India)

Of approximately 80 spices grown in different parts of the world, more than 50 are grown in India. This makes India an excellent case study for research; the spices that India produces and offers in abundance include pepper, ginger, turmeric, cardamom, fennel, cumin, dill, coriander, cinnamon, clove, nutmeg/mace, celery, fenugreek, and carom seeds. Spices used in the culinary, medical, and foodservice sectors, as well as in pharmaceuticals, depend on the plants’ leaves (curry leaf), buds (clove), bark (cinnamon), rhizomes (ginger, turmeric), berries (black pepper), seeds (cumin), or even flower stigma (saffron).

In Ayurveda, a conventional and ancient medicine system, many Indian kitchen spices are used and applied as immune-boosting ingredients; these spices deliver a wide array of health benefits to consumers’ various needs. Spices of Indian origin have flexible properties and are commonly used from the kitchen to the clinic. For instance:

- Cumin seeds have been used to treat colic pain, abdominal discomfort, flatulence, deficient lactation, piles (hemorrhoids), and worm infestation [25].
- Turmeric rhizomes have traditionally been used for skin diseases, blood impurities, coughs, asthma, insufficient lactation, obesity, and diabetes [25].
- Ginger and garlic are widely used spices in every household and are cultivated throughout India, particularly along the Western Ghats. Its rhizomes have traditionally been used to treat anorexia, colic pain, coughs, asthma, piles, pyrexia, and rheumatic diseases. Garlic bulbs were also used as medicine for rheumatism, colic pain, worm infestations, anorexia, and obesity [25].
- Cardamom and cinnamon have been used to cure bad breath, vomiting, excessive thirst, weakness, pyrexia, and burning sensations [25]. Cinnamon has also been found to have pharmacological, medicinal, digestive, stimulant, antibacterial, astringent, antioxidant, and antinociceptive properties [25].
- Pungent cloves and black pepper have been used to treat dental and oral diseases, throat infections, coughs, asthma, hiccoughs, colic pain, and anorexia, pyrexia obesity, and liver disease [25].
- Bay leaves have traditionally been used for urinary, pyrexia, rheumatic, and anorectic disorders [25].
- Curry leaves have traditionally treated diabetes, dysentery, anorexia, and flatulence [25].
- Nutmeg fruits and seeds have traditionally been used to treat dysentery, sexual disorders, and weakness.
- Mustard seeds have been used in treating skin diseases, dental disorders, worm infestations, obesity, dry skin, and anorexia.
- Various anise-scented spices have been used medicinally. Carom seeds have been used for flatulence, anorexia, diarrhea, colic pain, abdominal distress, flatulence, worm infestations, chronic fevers, and asthma. Star anise has historically been used for treating abdominal pain, flatulence, worm infestations, persistent fevers, and asthma. Fennel seeds have been used to treat colic pain, anorexia, difficult labor, and deficient lactation [25].
- Saffron flowers were used to treat bleeding disorders, vertigo, inflammation, skin and eye diseases, and headaches [25].

During the COVID-19 pandemic, the medicinal properties of spices are becoming increasingly recognized. This can perhaps be attributed to uncertainties about the coronavirus’s genetic mutations and strains, as well as the lack of any effective vaccines thus far. The resulting panic has incited people to try home remedies or natural medicines to boost their immunity. This can be one reason for the steady increase in spice exports in the global spice market since mid-June 2020. This moment could be used as an opportunity to enhance opportunities for spice tourism through effective marketing. Notably, spices are an integral part of many traditional medicines and wellness treatments that are now in high demand in medical tourism.

### Approach 2: Rural and agricultural tourism

Spices constitute many countries’ agricultural commodities, and they are essential to the culinary industry. Spice tourism is a type of “agro-tourism” that allows guests to acquire expertise and knowledge of various spices while exploring practices surrounding their cultivation and enjoying their fragrances. As an underdeveloped sub-sector of agro-tourism, attractions in spice tourism operate in the same context as similar tourism forms. However, it is likely a type of tourism that most tourists have never previously seen nor experienced. “At least 60 species of spice and herbs with its special value, in which these species have opportunities to conceived as tourism” [31].

Spice tourism can also contribute to the development of sustainable rural tourism [31]. Rural tourism is a viable tool for supporting sustainable development under several developmental constraints. Rural tourism offers beautiful sceneries, tranquil experiences, and fresh air and water, as well as insights into exciting traditional ways of life, cultures, and heritage. Studies on rural tourism have been carried out in numerous regions globally and have collectively noted its essential role in regional development. Research has also reported that various agriculturally productive areas have already been transformed into tourism settlements. Given agricultural conservation, preservation, and protection [4], it is essential to formulate sustainable rural development strategies. Spice tourism could offer a wide array of products and attractions to areas hosting rural tourism; the possibilities range from the study and production of spices, tourist accommodation in farmhouses, picnic areas at spice farms, spice vendor stalls, to restaurants that offer cuisine made from local spices.

### Approach 3: Food tourism

Traditional foods and ancient foodways are deeply rooted in many cultures’ traditional lifestyles and form essential parts of food-associated tourism [32]. Spices and herbs play a significant role in culture and often serve as nations’ symbols (black pepper for India). Since ancient times, spices have been used for cooking and medicine [33]. As many possess bioactive antimicrobial compounds, they have been valued for their ability to preserve foods and for their medicinal benefits [34]. Many customs involving spices are integral parts of both local and global culinary traditions, and the continued use of spices (in terms of intensity and type) depends on culinary traditions—the potential of spice tourism as a subsector within the framework of food tourism [35].

### Approach 4: Heritage tourism

The use of herbs and spices has been documented since ancient Egypt, and they have been used for centuries in India and China. “Exploration, trade, and wars were related to the search for spices contributing to the establishment of spice trading routes. Through colonization and migration space, cultivation was spread around the world” [14]. There are two main ways in which spices could be used as a cultural heritage attraction: (1) by relating spices to food and cultural attractions as designated by UNESCO, as is the case of Zanzibar and (2) by positioning spices within ancient trade and heritage, as in the Spice Route Project in Kerala, India. The first category has been discussed in the “Approach 3: Food tourism” section. As for the second strategy, UNESCO established a heritage tourism project incorporating the ancient spice trade in mid-2019 in Kerala, India.

The Spice Route Project by Kerala Tourism guides tourists and travelers along a 2000-year-old trade route, one of many that connected ancient South India with approximately 30 countries worldwide. UNESCO recently recognized the project, and the Netherlands, Portugal, Myanmar, Britain, Iraq, Afghanistan, Iraq, Indonesia, China, and Iran all consented to these initiatives. Archeological excavations at Muziris (a port town in Kerala) unearthed concrete evidence that Muziris had traded with the West; these discoveries kicked off the Spice Route Project. This project aims to educate people on the history of the region and open opportunities for east-to-west travel. This has the potential to strengthen international relations and help promote shared heritage through tourism.

## Spice tourism in the context of COVID-19

The health benefits and immunity-boosting properties of spices could be exploited to reformulate spice tourism. Many Indian kitchen spices are regularly used as immune-boosting ingredients in traditional and Ayurvedic medicine; these spices continue to offer ample health benefits for consumers’ various needs. COVID-19 has been found to affect those with weaker immune systems, particularly the elderly. The immune system is based on live microbiota that shields the body against infectious illnesses. Several studies suggest that plant-based foods enhance and strengthen protective microbial populations and gut microbiome’s general health, comprising 85% of the human immune system [36].

Herbs and spices are renowned for boosting immunity. Immunity-building functional foods such as garlic, turmeric, ginger, ashwagandha, herbal tea, and intensely antioxidant-rich fruit have the potential to enhance immunity, and that people should more greatly incorporate these products into their diets [37].

**Fig. 2:**
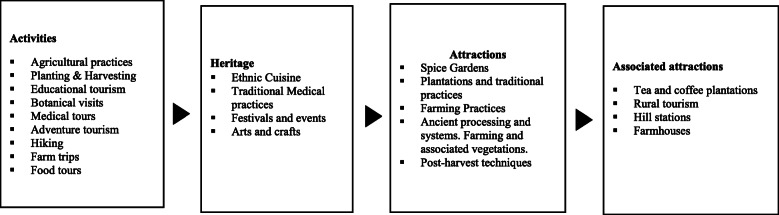
Positioning spice tourism as SIT (Source: Authors)

In conjunction with these epidemiological survey, the demand for Indian spices skyrocketed in mid-2020, with spice sales increasing 23%, or by $359 million, in June of 2020 [38, 39] Furthermore, Kadha, a popular drink made of herbal extracts and nutritious spices, evolved as a drink of choice during the COVID-19 pandemic due to its immune-enhancing properties. The Indian government, exploiting this trend to propel global marketing in AYUSH (Ayurveda, Yoga & Naturopathy, Unani, Siddha, and Homeopathy) and commercial exports. This juncture serves as an excellent opportunity to deploy spice tourism as an emerging SIT form [14] (see Fig. 2).

## Concluding remarks: Strategies for product development

**Fig. 3:**
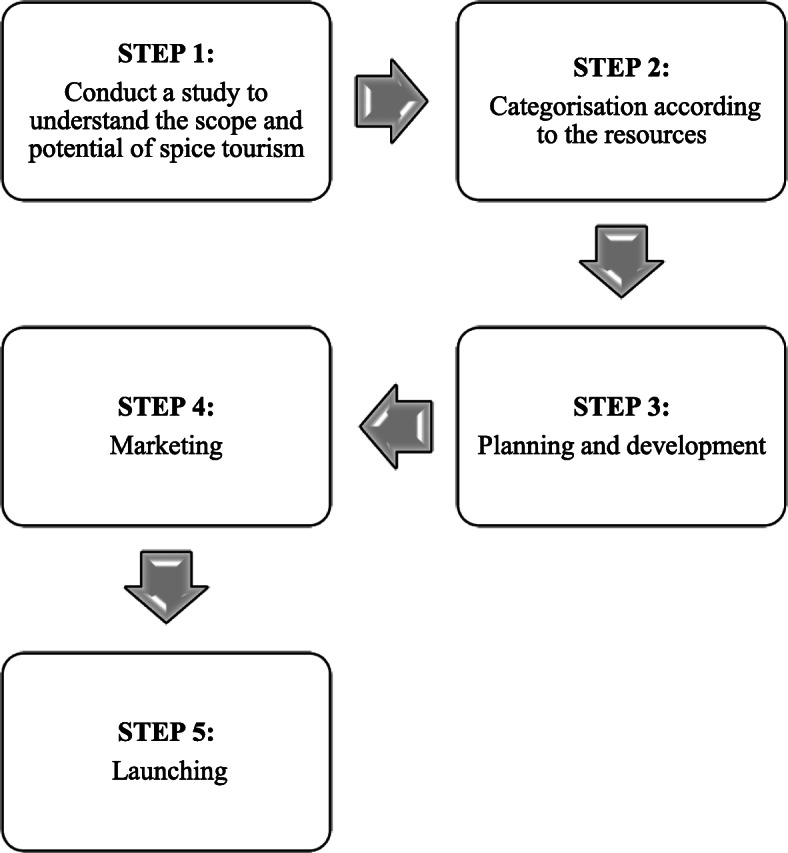
Product development strategies for spice tourism

This paper recommends several product development strategies for spice tourism (Fig. 3). First, identifying the diversity of spices and herbs should constitute their development as a tourism product. Second, the spices should be categorized based on their various functions, ranging from economic to cultural. Third, specific activities and their duration should be identified. The last stages will consist of marketing and launching these products and activities [14].

There are many opportunities for developing or reformulating spice tourism, especially in light of growing demand. Many countries already have spice tourism projects or have identified their potential. These projects can be centered on the specific local spices growing in a region (e.g., saffron tourism in Kashmir, India) or highlight many spices collectively in tourist attractions (e.g., the Spice Park in Kumaly, Kerala, India). In whatever form they take, infrastructure development and adequate marketing are crucial moving forward.

## Data Availability

All data and materials have been presented in the manuscript.
